# Nurses’ perception of the nursing process and its relationship with leadership

**DOI:** 10.1590/0034-7167-2023-0371

**Published:** 2024-04-22

**Authors:** Karen Ariane Bär, Bianca dos Santos Lima, Gicelle Morais Martelle, Silvana Cruz da Silva, Margarida Reis dos Santos, Regina Gema Santini Costenaro

**Affiliations:** I Universidade Franciscana. Santa Maria, Rio Grande do Sul, Brazil; II Escola Superior de Enfermagem. Porto, Portugal

**Keywords:** Nursing Research, Nursing Process, Patient Care Teams, Leadership, eHealth Strategies, Investigación en Enfermería, Proceso de Enfermería, Grupo de Atención al Paciente, Liderazgo, Estrategias de eSalud, Pesquisa em Enfermagem, Processo de Enfermagem, Equipe de Assistência ao Paciente, Liderança, Estratégias de eSaúde

## Abstract

**Objectives:**

to describe Nurses’ perception of the Nursing Process and its relationship with leadership.

**Methods:**

action research conducted between September/2021 and April/2022 with nurses from a medium-sized hospital in southern Brazil. The data investigated, one of the stages of the method, was collected using the Focus Group technique and submitted to Strategic Focus Analysis.

**Results:**

three categories emerged from the organized and analyzed data, namely: Nursing Process: a tool that qualifies nursing care; Conditions that weaken the Nursing Process; and Strategies that enhance the Systematization of Nursing Care.

**Final Considerations:**

the perception of the Nursing Process and its relationship with leadership are not always understood as complementary themes. Although they recognize that the Nursing Process is sometimes imposed as normative, nurses do not perceive the importance of the role of the leader, who is considered a key player in conducting and boosting the Systematization of Nursing Care.

## RESUMO

**Objetivos:**

descrever a percepção dos Enfermeiros sobre o Processo de Enfermagem e a sua relação com a liderança.

**Métodos:**

pesquisa-ação conduzida entre setembro/2021 e abril/2022 com enfermeiros de um hospital de médio porte do sul do Brasil. Os dados investigados, uma das etapas do método, foram coletados por meio da técnica de Grupo Focal e submetidos à Análise Focal Estratégica.

**Resultados:**

dos dados organizados e analisados resultaram três categorias, quais sejam: Processo de Enfermagem: ferramenta qualificadora da assistência de enfermagem; Condições que fragilizam o Processo de Enfermagem; e Estratégias que potencializam a Sistematização da Assistência de Enfermagem.

**Considerações Finais:**

a percepção de Processo de Enfermagem e a sua relação com a liderança nem sempre são apreendidos como temas complementares. Embora reconheçam que o Processo de Enfermagem é, por vezes, imposto como normativo, os Enfermeiros não percebem a relevância da função do líder, considerado ator-chave na condução e dinamização da Sistematização da Assistência de Enfermagem.

## RESUMEN

**Objetivos:**

describir la percepción de los Enfermeros sobre el Proceso de Enfermería y su relación con el liderazgo.

**Métodos:**

se trata de una investigación-acción realizada entre septiembre de 2021 y abril de 2022 entre enfermeros de un hospital de medio porte del sur de Brasil. Los datos investigados, una de las etapas del método, fueron recolectados mediante la técnica de Grupo Focal y sometidos al Análisis Focal Estratégico.

**Resultados:**

los datos organizados y analizados resultaron en tres categorías, a saber: Proceso de Enfermería como herramienta que cualifica los cuidados de enfermería; Condiciones que debilitan el Proceso de Enfermería; y Estrategias que potencian la Sistematización de los Cuidados de Enfermería.

**Consideraciones Finales:**

la percepción del Proceso de Enfermería y su relación con el liderazgo no siempre se toma en cuenta como tema complementario. Aunque los Enfermeros reconocen que el Proceso de Enfermería se impone a veces como normativo, no perciben la importancia del papel del líder, considerado como un actor clave para conducir y dinamizar la Sistematización de los Cuidados de Enfermería.

## INTRODUCTION

The Nursing Process (NP) is a tool that supports the nurse in the process of caring for the client being cared for and in documenting professional practice^(1)^. The NP organizes and systematizes care based on five interdependent and interrelated stages: data collection, nursing diagnoses, planning, implementation and nursing evaluation^(1,2,3)^.

Based on theoretical references, it encourages critical-reflective thinking and communication between the nursing team and between the nursing team and other health professionals^(4,5)^, as well as continuity and individuality of care, and supports nurses in decision-making. This can be optimized and qualified with the support of technological tools, such as computerized forms or instruments that enable faster and more effective nurse-user/ family interaction. A study shows important advantages related to the computerization of nursing care, emphasizing that computerization qualifies, optimizes and expands the achievement of expected health results, as well as enabling information sharing and collaborative work between health professionals and users^(6)^.

Another study^(7)^ shows that eHealth strategies, unlike traditional care methods and processes, provide more concise, objective and reliable information to the professionals involved in health care. The same study assures that the new eHealth strategies favor the planning of priorities and the establishment of goals and collegiate actions. However, these new methodological and technological approaches alone do not guarantee the necessary advances in health/nursing. Leadership needs to be discussed in parallel in order to enable increasingly constructive and collaborative paths between work teams.

However, studies that highlight the Nursing Process and its relationship with leadership are scarce, despite recognizing that the leader plays a key role in the different professional scenarios. Studies^(8,9,10)^ recognize that technological investments and new methodological approaches associated with the health work process are not enough if they are not accompanied by strong leadership. The leader plays an influential role in this process, directing goals and actions in order to achieve the desired results. Based on the collegiate agreements and outlines, the research question was: what are nurses’ perceptions of the Nursing Process and its relationship with leadership?

## OBJECTIVES

To describe nurses’ perceptions of the nursing process and its relationship with leadership.

## METHODS

### Ethical aspects

The recommendations of National Health Council Resolution 466/2012 were followed. The project was submitted to and approved by the Research Ethics Committee and written informed consent was obtained from all the individuals involved in the study. The study participants were identified throughout the text by the letter N (Nurse) plus a numerical number, according to the order in which they spoke.

### Theoretical-methodological framework

The theoretical framework used was the assumptions of the Systematization of Nursing Care, used as a method for planning, organizing, directing and operationalizing the Nursing Process, as a tool for inducing better nursing practices.

### Type of research

This is an action research project^(11)^ this study aims to describe nurses’ perceptions of the Nursing Process and its relationship with leadership, with a view to implementing a computerized nursing record in a pediatric inpatient unit. The aim was to develop, in addition to an investigative path, improvements in the care practices of nursing professionals, through awareness-raising meetings, with a view to developing and implementing the Nursing Process in all hospitalization units. The Consolidated Criteria for Reporting Qualitative Research (COREQ) criteria were taken into account throughout the research^(12)^.

### Methodological procedure

#### Study scenario

Conducted in interdependent steps, action research^(11)^ in this study, the process was based on sequential and complementary stages, namely: identifying the demand in the context, investigating relevant data, analyzing and giving meaning to the data investigated, outlining prospective intervention-action strategies and, finally, evaluating the implemented product in order to highlight the expected progress.

Stage 1: Identification of the demand in the context - Based on dialogue with nursing managers at a hospital in the interior of southern Brazil, there was a demand associated with the computerization of the Nursing Process, starting with the Nursing History, in order to enable greater engagement and satisfaction among professionals in relation to its implementation in the different hospitalization units.

Stage 2: Investigation of relevant data - Eight focus group meetings were held with nurses who work directly or indirectly in a Pediatric Inpatient Unit, with the aim of finding out about their perceptions and experiences associated with the Nursing Process and their opinion on its relationship with the exercise of leadership. The focal meetings were held according to a specific schedule, which included themes, days, times, duration, the name of the coordinator and monitors, as well as criteria for participation.

Stage 3: Analysis and meaning of the data investigated - This phase was conducted simultaneously with the focus groups, based on the Strategic Focus Analysis, the design of which followed the steps proposed in a previous study. In this step, the aim was to identify and analyze the weaknesses, potential and prospective strategies for inducing the Nursing Process.

**Figure 1:**
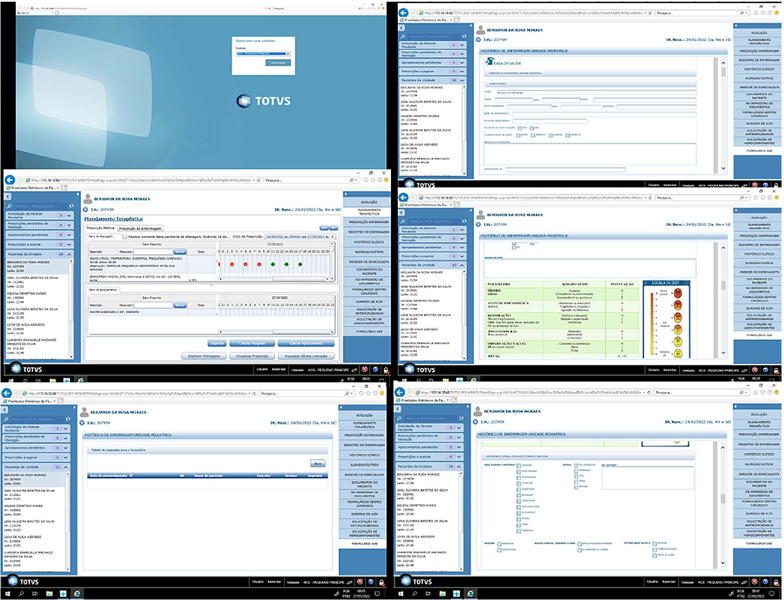
Layout of the computerized Nursing History, Santa Maria, Rio Grande do Sul, Brazil, 2022

Stage 4: Outlining prospective intervention-action strategies - Based on the analysis and significance of the data investigated, the development of a computerized Nursing History tool for a Paediatric Inpatient Unit was envisaged, as proposed by the hospital’s managers. Construction began on the pediatric physical examination scripts that would make up the computerized nursing history, as well as the product layout, as shown in figure 1. These scripts were drawn up based on the needs affected by the children admitted to this pediatric unit. At the same time, systematic meetings were held to study, discuss and raise awareness of how to understand and implement the Nursing Process, and more specifically the computerized Nursing History, with the support of a technical professional from the IT area, as well as specific advisory services.

Stage 5: Evaluation of the implemented product - In this phase, feedback was given on the development and implementation of the computerized Nursing History in the TOTVS system, a company accredited by the hospital in question.

In this study, only the results of the second step of the action research will be presented and discussed, relating to the description of nurses’ perceptions of the Nursing Process and its relationship with leadership.

#### Study participants

The participants in this study were 14 nurses with at least two years’ professional experience at the hospital. This study included nurses who work directly or indirectly in the Pediatric unit of a teaching hospital accredited by the Unified Health System. This unit has 16 beds for children aged between one month and 14 years. The hospital in question is a reference for 32 other municipalities in the region. Nurses who, for whatever reason, were absent from work at the time of data collection, or those who were unable to take part in the schedule of meetings previously made available, were excluded from the study. Based on these criteria, two nurses were excluded, one due to pregnancy leave and the other for health reasons. Of the nurses included, three had been working for more than six years and the others between two and five years.

#### Data collection and organization

The data was collected between September/2021 and April/2022, using the Focus Group technique, characterized by a discussion group that talks about a particular topic, experienced and shared through common experiences^(13)^. This technique was chosen because it enables broad discussions on a particular topic, in this case, the Nursing Process and nurse leadership. The interaction, discussion and prospecting of strategies between group participants is one of the main characteristics of this technique.

The meetings/focus groups were moderated by a researcher, who took on the role of coordinating the debates, and an observer (a postgraduate student). The observer supported the coordinator throughout the process and was responsible for recording the collective speeches, the notes and the dynamics of the meetings. A total of eight meetings/focus groups were held, lasting a maximum of 90 minutes. In each of the meetings, the potential, weaknesses and strategies associated with leadership and the dynamization of the Nursing Process were considered.

The first five meetings focused on the stages of the Nursing Process and the last three focused on discussions about the role of leadership and outlining prospective strategies for the effective development of the Nursing Process. At the beginning of each meeting, the moderator presented a reflective summary of each of the stages of the Nursing Process, based on scientific evidence on the subject. The participants were then encouraged to share their perceptions and experiences, which led to intense discussions, which were expanded in the light of references from the proposed thematic area. At the end of each meeting, an integrated summary of the discussions was produced, which was outlined by the moderator and validated by all the participants.

#### Data analysis

Data analysis began with a comprehensive summary of each of the meetings, as suggested by the Strategic Focus Analysis (SFA)^(13)^, which involves the critical and reflective participation of the participants. Finally, the researchers carried out a theoretical analysis of the synthesis of each of the eight meetings, with the aim of broadening the phenomenon under investigation, in this case, the Nursing Process and its relationship with leadership. In this process, they considered the potential and weaknesses, as well as the challenges, opportunities and, above all, strategies that indicate improvements. In addition to the descriptive summary of each of the meetings, the participants’ speeches were recorded and transcribed in order to broaden and deepen the subject under investigation.

## RESULTS

The data organized and analyzed resulted in three categories: Nursing Process: a tool that qualifies nursing care; Conditions that weaken the Nursing Process; and Strategies that enhance the systematization of nursing care.

### The Nursing Process: a tool for qualifying nursing care

It was clear from the summary of all the meetings held that the Nursing Process has significance and relevance in nurses’ daily practice. This process, however, is not always decipherable and palpable in its theoretical and practical conception for some nurses. The participants, however, were unanimous in stating that the Nursing Process is characterized as a guiding and qualifying tool for nursing care, although they showed difficulties in making this process more dynamic with the nursing technicians.

*It is an important tool for the work of the nursing team and guides the nursing technician in the practice of patient care. The nurse manages and guides the team’s care, but I find it difficult to coordinate this process with the nursing technicians.* (N1)

*How can nursing care be carried out without a clear methodology to guide the team’s work? The nurse is the team leader and responsible for this process.* (N5)

The Nursing Process was associated/compared several times with the SNC, even though in essence the participants wanted to express the same idea. Although this tool has been confused with the SNC, the participants recognize it for its potential to induce autonomy, appreciation and scientific in the nurse’s professional work.

*The SNC is valid for organizing the practice of nursing care in patient care. This process shows that nursing does, gives autonomy, does not depend on the doctor to prescribe.* (N2)

*I think it’s important for institutional organization. It gives nurses more autonomy and value. It guarantees individualized care, based on nursing care planning.* (N13)

Although it is considered relevant, pertinent and induces better nursing practices, the Nursing Process is not yet a reality in all health units/institutions, especially in municipalities far from large centers. This perception was evident in the speech of one nurse, in particular, when he mentioned that the SNC should be routine in any and all institutions, but he realized that progress is slower in small towns.

*SAE is of the utmost importance. It is necessary. It’s the nurse’s main instrument when dealing with the patient, and it should be routine in any institution. But I understand that for those of us from further inland, everything is slower.* (E4)

The general summary of the focus meetings showed that nurses have basic and superficial notions about the Nursing Process and its relationship with leadership, even though they demonstrated its potential for improving care. Some of the participants made a huge effort to create prospective movements for its implementation in all units and institutions.

### Conditions that weaken the Nursing Process

The synthesis of the various focal meetings revealed conditions that weaken the operationalization of the nursing process in hospitalization units, especially with regard to the motivation of the work team and the difficulties related to the paediatric physical examination, considered an indispensable component in taking the nursing history, as expressed by:

*I confess that I find it difficult to carry out the stages of the nursing process. The physical examination in pediatrics is very specific and I feel very insecure.* (N2)

*I try to value and carry out the Nursing Process, but I’m afraid of doing something wrong, especially in the physical examination. I always have doubts about how to work on these issues with the team.* (N13)

At various times, the participants compared the different hospitalization units, showing that in some the teams are not very collaborative in implementing the Nursing Process, as follows:

*Here everyone writes the prescription and everything works well, but in other units there is a lack of involvement. It seems that the team has no interest.* (N2)

*In my unit, everyone got involved, but I need to continually reiterate the importance of checking the nursing prescription*. (N3)

*In the maternity ward, I manage to develop the SNC on a daily basis, unless the unit is very busy and has a lot of demands. But I notice that in many units this process is not organized and only problems are solved. It seems that the professionals haven’t yet understood its importance.* (N6)

It is clear from the statements above that the Nursing Process organizes the work environment and systematizes nursing care, with the possibility of transcending the disease focus. In those units where the Nursing Process is not systematized, the focus of care is on “solving problems”. It is clear, therefore, that the Nursing Process is capable of defocusing the nurse’s work on “problems”, inducing them to plan and prospect strategies that lead to the prioritization of conducts, based on prospective decision-making.

It was also noted that for some nurses the stages of the Nursing Process are considered redundant, especially the nursing prescription. This thinking may be associated with a limited and/or superficial understanding of its real meaning, confusing the prescription of nursing interventions with the medical prescription or considering that the medical team should prescribe nursing interventions.

*I don’t usually write nursing prescriptions, because I think it’s redundant, since the medical team ends up writing the same prescriptions. Which is still a concern, because it feels like we’re not doing our job any more.* (N8)

There was also an insufficient number of nurses to cope with the daily work demands. The position related to “solving problems” may be associated with the impossibility of carrying out all the stages of the Nursing Process, since in these cases care is perceived as fractioned and reduced to prioritizing “problems”, as expressed by:

*There are too few nurses here to do everything. At weekends, I’m always called in to solve problems, especially venipuncture or the transfer of children who aggravate the health situation. I spend all my time solving problems.* (N3)

*Sometimes they call me just to solve problems, but I always say that there should be more nurses. Then I get very divided.* (N1)

The weaknesses associated with the operationalization of the Nursing Process in practice must be analyzed from different perspectives and under different conditions. If, on the one hand, there is a limited and superficial theoretical-methodological understanding of the Nursing Process, on the other there is a certain trivialization and/or weak leadership on the part of nurses who prefer to remain in a position of comfort and security.

### Strategies that enhance the systematization of nursing care

Several strategies were listed by the study participants that enhance the Nursing Process and the systematization of nursing care in hospital inpatient units. In addition to expanding the spaces for discussion on the theoretical and methodological concepts of the Nursing Process and leadership, the participants suggested working collaboratively with other health professionals in order to boost initiatives, share achievements and enable comprehensive care for patients and their families.

*We need more spaces to discuss the nursing process and leadership. Nurses should study the subject more. Many things need to be improved to give it a new meaning.* (N3)

*As it says, the nursing process is a nursing process, but we need to work as a team with other health professionals. We need to learn from each other and value what each professional does well and then do it even better.* (N6)

Another important strategy mentioned was the implementation of a specific nursing record for the pediatric inpatient unit, considering that each unit has its own particularities. The participants also recognized the need to develop functional electronic documentation systems that facilitate decision-making support on diagnoses, expected results and nursing interventions, and that make it possible to reduce the time needed to produce nursing records.

*We need technologies that are easy to access, objective and functional, which makes it easier for nursing professionals and the healthcare team to understand and operate them.* (N6)

*I understand that we need tools that cover all the stages of the nursing process. The implementation of a computerized tool favors systematized and safe care. The development and implementation of computerized tools was planned in the strategic planning, but we haven’t been able to move forward. We need support to leverage this process*. (N11)

*We need tools to support care that don’t take up so much of the nurse’s time. Computerizing the Nursing Process qualifies management and optimizes care. But it must not become a mechanical process. Nurses need to guide their teams in a critical and informed way, with clinical knowledge.* (N13)

Other participants also pointed out that it is necessary to consider the specific demands of each hospitalization unit, based on spaces for dialogue with the leaders directly involved in nursing care. It was clear that nurses want to be heard and integrated into strategic decision-making and, from this point of view, denoted that the development of a system for computerizing clinical nursing documentation must be built with the participation of all those involved, which contributes to nurses’ adherence to its use.

*The tools need to include all the data needed to carry out the nursing process in each of the hospitalization units. It is essential that the technological tools contain important information for understanding the patient’s clinical condition and provide a basis for the other parts of the nursing process. Being able to discuss and talk about this subject has even changed my willingness to carry out this process, which is so important for the advancement of nursing.* (N7)

In short, the possibility of developing a Nursing Process with all the stages computerized and in a participatory and collaborative way rekindled the “willingness to carry out this process” in several participants. This demonstrates the importance of producing technologies in cooperation with the professionals directly involved in the operationalization of the Nursing Process, based on demands previously identified and agreed with local leaders. From this point of view, the collaborative production of technologies has become a more effective alternative to the traditional and hegemonic models of project management and production.

## DISCUSSION

The results of this study show a certain confusion between the Systematization of Nursing Care and the Nursing Process, which is not uncommon in other scenarios. In this sense, the focus groups enabled broader discussions and the sharing of knowledge and practices about the purpose of these theoretical-methodological tools, as well as broadening their relationship with the theme of leadership, which is essential for the effective implementation of the Nursing Process.

Studies^(14,15,16)^ have previously expressed concern about the lack of clarity between what is meant by the Nursing Process and the Systematization of Nursing Care. For these authors, the SNC cannot be understood as synonymous with the Nursing Process, at the risk of reducing and relativizing its primary function. The SNC, unlike the Nursing Process, does not take place in stages, but is based on pillars, methods, personnel and instruments that systematize and organize the management and organization of nursing care, in cooperation with other health professionals.

Throughout the research process, the nurses repeatedly referred to their exhausting work routine. The Nursing Process was generally treated as a “hard, exhausting, demotivating process”. Based on this finding, questions and reflections were raised among the participants, such as: What are the main reasons that justify the Nursing Process as “hard, exhausting, demotivating”? How can nursing care be systematized in order to make the work routine more dynamic and flexible? These and other questions were asked in order to broaden the theoretical-methodological perception of the Nursing Process, especially in terms of its benefits, advantages and prospective outlines, associated with the role of local leadership.

A study^(17)^ revealed, from this perspective, that difficulties related to the operationalization of the Nursing Process may indicate lack of knowledge, lack of appropriation by the team or obstacles related to leadership. This same study also mentioned that not using a scientific method to support the work process interferes with the quality of management and care as well as professional (in)satisfaction. This thought is corroborated by the participants in this study, who mentioned that in certain units “only problems are solved”. Systematizing nursing care means, from this point of view, establishing priorities and goals and ensuring leadership that aggregates and drives new knowledge.

Another element repeatedly emphasized by the participants in this study is associated with the argument “lack of time” to carry out the Nursing Process, as if it were something external and/or imposed by higher authorities. Based on this thought, several reflections emerged which led the participants to think that the “lack of time” may be linked to the inoperability of the Nursing Process in practice and to the lack of effective leadership, considering its potential to dynamize and induce organization and planning.

As Studies^(18,19,20)^ have shown that “lack of time” can be considered a limiting condition to the implementation of the Nursing Process, others have shown that the Nursing Process favors the optimization of time, the sharing of knowledge and the implementation of a plan of goals previously induced by the team leader^(21,22)^. From this perspective, it can be seen that the Nursing Process requires greater theoretical and methodological depth on the part of local leaders, but also on the part of the nursing teams, in order for it to be seen as elementary and primordial when it comes to providing unique and multidimensional nursing care.

In the meetings/focus groups, the participants mentioned several times that technologies to support nursing management and care are often developed without the proper participation of the professionals directly involved in the care. With this in mind, a study has shown that the development of digital tools to support the operationalization of the Nursing Process should consider the participation of local leaders and work teams in the different stages, as well as being based on specific taxonomies^(23)^. Another study reinforces this thinking by emphasizing that the permeation of digital technologies is not changing the organization of teamwork, but it is changing the nature of teamwork, in which leadership plays an increasingly important role^(24)^.

Digital technologies and processes are increasingly in demand, especially in the post-pandemic period when different sectors and areas of knowledge have expanded their digital scope. In nursing, this process is no different and tends to grow more and more, such as the recent regulation of Telenursing, under Resolution 696/2022, which establishes the guidelines for nurses to work in Digital Health, both in the public and private sectors^(25)^.

It is not enough, however, to advance in terms of technological innovation. Studies have shown that digitalization and technological innovation need to go hand in hand with leadership. Developing leadership skills in the digital age, good practices for leading and conducting virtual teams are currently the main findings in the different areas of knowledge^(26,27)^. In the development of a digital/technological culture, in which all the members of a team feel they are protagonists and co-responsible, leaders are drivers and key players.

### Study limitations

The limitations are associated with the fact that this study was carried out with nurses from just one small hospital in southern Brazil, which makes generalizations impossible.

### Contributions to the field of Nursing

The main contribution of this study to the nursing field is associated with the demonstration that the Nursing Process, whether computerized or not, needs to be promoted by nurse leaders, in order to promote collegiate cooperation with team members. It is not enough to invest in technological innovation or in computerizing/digitizing the Nursing Process. At the same time, it is essential to invest in nursing leaders who can take the lead, innovating and translating new knowledge into professional practice.

## FINAL CONSIDERATIONS

The perception of the Nursing Process and its relationship with leadership are not always seen as complementary themes. Although they recognize that the Nursing Process is sometimes imposed as normative and in an uncritical way, nurses do not perceive the importance of the role of the leader, who is considered a key actor in conducting and boosting the Nursing Process.

In short, it shows that nurses have a basic understanding of the Nursing Process and its relationship with leadership. However, there is a need to expand studies and discussions to differentiate the Nursing Process from the Systematization of Nursing Care, in order to clarify conceptions, demystify beliefs and boost initiatives for a better understanding of the benefits of this tool and its direct relationship with leadership.
